# Supplementary material for: Genetic Localization and Homologous Genes Mining for Barley Grain Size
Source: Int J Mol Sci. 2023 Mar 3;24(5):4932. doi: 10.3390/ijms24054932 (PMC10003025; doi:10.3390/ijms24054932)
Supplement: Supplementary file 1 [file ijms-24-04932-s001.zip › ijms-2216101-supplementary.pdf]

Table S1. Genetic correspondence between barley and other cereal genes controlling grain development.

Orthologs	Collinearity	Morex_ID	Accession number	Description	Reference
HvGSN1		HORVU.MOREX.r3.1HG0008520	Os05g0115800	Dual specificity phosphatase	[47]
HvRSR1		HORVU.MOREX.r3.1HG0012250	Os05g0121600	AP2-like ethylene-responsive transcription factor	[48]
HvAPG	*yes*	HORVU.MOREX.r3.1HG0018510	Os05g0139100	Basic helix-loop-helix transcription factor	[93]
HvPPKL2	*yes*	HORVU.MOREX.r3.1HG0019870	Os05g0144400	Serine/threonine-protein phosphatase	[73]
HvTaPGS1	*yes*	HORVU.MOREX.r3.1HG0021280	TraesCS1D02G094000	Transcription factor basic helix-loop-helix	[94]
HvSRS3	*yes*	HORVU.MOREX.r3.1HG0023370	Os05g0154700	Kinesin-like protein	[95]
HvGSK2		HORVU.MOREX.r3.1HG0030650	Os05g0207500	Kinase family protein	[96]
HvAGSW1		HORVU.MOREX.r3.1HG0036390	Os05g0323800	ABC1-like kinase	[97]
HvBSK2	*yes*	HORVU.MOREX.r3.1HG0052470	Os10g0571300	Serine/threonine-protein kinase BSK2	[49]
HvSM1	*yes*	HORVU.MOREX.r3.1HG0058550	Os05g0389000	AP2-like ethylene-responsive transcription factor	[52]
HvLAC1		HORVU.MOREX.r3.1HG0073500	Os05g0458600	Laccase	[98]
HvSLG		HORVU.MOREX.r3.1HG0076820	Os08g0562500	HXXXD-type acyl-transferase family protein	[53]
HvO2		HORVU.MOREX.r3.1HG0078350	Zm00001eb301570	Basic-leucine zipper (BZIP) transcription factor family	[99]
Hvmrp1-1		HORVU.MOREX.r3.1HG0081040	Zm00001eb354390	MYB-related transcription factor	[100]
Hvincw1-1		HORVU.MOREX.r3.1HG0087260	Zm00001eb242820	Cell wall invertase	[57]
HvIKU2-1		HORVU.MOREX.r3.1HG0088480	At3g19700	Leucine-rich repeat receptor-like protein kinase	[72]
HvEF3.2	*yes*	HORVU.MOREX.r3.1HG0095050	Os01g0566100	Early flowering 3	[101]
HvSDG725		HORVU.MOREX.r3.2HG0096180	Os02g0554000	Histone-lysine N-methyltransferase	[58]
HvFZP	*yes*	HORVU.MOREX.r3.2HG0114260	Os07g0669500	Ethylene-responsive transcription factor	[102]
HvMADS87-1		HORVU.MOREX.r3.2HG0119930	Os03g0582400	AGAMOUS-like MADS-box protein	[103]
HvMADS87-2		HORVU.MOREX.r3.2HG0119950	Os03g0582400	AGAMOUS-like MADS-box protein	[103]
HvTaSus2-2B	*yes*	HORVU.MOREX.r3.2HG0124890	TraesCS2B02G194200	Sucrose synthase	[104]
HvDEP2		HORVU.MOREX.r3.2HG0125030	Os07g0616000	Erect panicle 2 protein	[105]
HvGW7	*yes*	HORVU.MOREX.r3.2HG0126960	Os07g0603300	Longifolia protein	[106,107]
HvGE	*yes*	HORVU.MOREX.r3.2HG0127090	Os07g0603700	Cytochrome P450 protein	[108]
HvBZR1	*yes*	HORVU.MOREX.r3.2HG0131380	Os07g0580500	BES1/BZR1 homolog 1	[109]
HvRPT2A-1		HORVU.MOREX.r3.2HG0139190	At4g29040	26S protease regulatory subunit	[110]
HvSPL13	*yes*	HORVU.MOREX.r3.2HG0142950	Os07g0505200	Squamosa promoter-binding protein	[7]
HvANT-1		HORVU.MOREX.r3.2HG0154350	At4g37750	AP2-like ethylene-responsive transcription factor	[111]
HvGIF1	*yes*	HORVU.MOREX.r3.2HG0166450	Os04g0413500	Cell wall invertase	[112]
HvTabas1-B1	*yes*	HORVU.MOREX.r3.2HG0167220	TraesCS2B02G313700	Peroxiredoxin	[113]
HvD11		HORVU.MOREX.r3.2HG0174520	Os04g0469800	Cytochrome P450 protein	[114]
HvCyclin-T1;3-1		HORVU.MOREX.r3.2HG0176090	Os11g0157100	Cyclin family protein	[115]
HvLABA1	*yes*	HORVU.MOREX.r3.2HG0182190	Os04g0518800	Cytokinin riboside 5'-monophosphate phosphoribohydrolase	[116]
HvEXPA10		HORVU.MOREX.r3.2HG0185850	Os04g0583500	Expansin	[117]
HvXIAO	*yes*	HORVU.MOREX.r3.2HG0186650	Os04g0576900	Non-specific serine/threonine protein kinase	[118]

Hvnkd1-1	*yes*	HORVU.MOREX.r3.2HG0187780	Zm00001eb073830	Zinc finger protein, putative	[119]
HvSMG2	*yes*	HORVU.MOREX.r3.2HG0188270	Os04g0559800	Protein kinase	[120]
HvSPL33		HORVU.MOREX.r3.2HG0194480	Os01g0116600	Elongation factor 1-alpha, putative	[121]
HvZm-INVINH1-1	*yes*	HORVU.MOREX.r3.2HG0196570	Zm00001eb072630	Invertase inhibitor	[122]
HvZm-INVINH1-2		HORVU.MOREX.r3.2HG0196590	Zm00001eb072630	Invertase inhibitor	[122]
HvZm-INVINH1-3		HORVU.MOREX.r3.2HG0196600	Zm00001eb072630	Invertase inhibitor	[122]
HvZm-INVINH1-4		HORVU.MOREX.r3.2HG0196610	Zm00001eb072630	Invertase inhibitor	[122]
HvZm-INVINH1-5		HORVU.MOREX.r3.2HG0196620	Zm00001eb072630	Invertase inhibitor	[122]
HvZm-INVINH1-6		HORVU.MOREX.r3.2HG0196630	Zm00001eb072630	Invertase/pectin methylesterase inhibitor family protein	[122]
HvAP2		HORVU.MOREX.r3.2HG0204770	At4g36920	AP2-like ethylene-responsive transcription factor	[123]
HvFLO2	*yes*	HORVU.MOREX.r3.2HG0205380	Os04g0645100	Clustered mitochondria protein	[124]
HvZmAFL4	*yes*	HORVU.MOREX.r3.2HG0210050	Zm00001eb066270	B3 domain transcription factor	[125]
HvRAG2		HORVU.MOREX.r3.2HG0212810	Os07g0214300	Dimeric alpha-amylase inhibitor	[126]
HvBSK3	*yes*	HORVU.MOREX.r3.2HG0214020	Os04g0684200	Kinase family protein	[127]
HvCKX2-1		HORVU.MOREX.r3.3HG0236930	At2g19500	Cytokinin oxidase/dehydrogenase	[76,128]
HvMADS87-3		HORVU.MOREX.r3.3HG0244110	Os03g0582400	AGAMOUS-like MADS-box protein	[103]
HvGn1a/CKX2-1	*yes*	HORVU.MOREX.r3.3HG0244570	Os01g0197700	Cytokinin oxidase/dehydrogenase	[129]
HvGn1a/CKX2-2		HORVU.MOREX.r3.3HG0244590	Os01g0197700	Cytokinin oxidase/dehydrogenase	[129]
HvRDD1	*yes*	HORVU.MOREX.r3.3HG0255050	Os01g0264000	Dof zinc finger protein	[130]
HvFBK12-1	*yes*	HORVU.MOREX.r3.3HG0255740	Os03g0171600	Kelch repeat-containing protein	[131]
HvHB46	*yes*	HORVU.MOREX.r3.3HG0275220	Zm00001eb158680	Homeobox associated leucine zipper protein	[132]
Hvemp16	*yes*	HORVU.MOREX.r3.3HG0279170	Zm00001eb359970	Pentatricopeptide repeat-containing protein	[133]
HvD61		HORVU.MOREX.r3.3HG0285210	Os01g0718300	Receptor kinase	[66]
HvBG1-1	*yes*	HORVU.MOREX.r3.3HG0294270	Os03g0175800	Protein BIG GRAIN 1	[134]
HvABI5		HORVU.MOREX.r3.3HG0300770	At2g36270	BZIP transcription factor	[77]
HvDET2	*yes*	HORVU.MOREX.r3.3HG0301780	At2g38050	3-oxo-5-alpha-steroid 4-dehydrogenase family protein, expressed	[82]
HvABI3/HvVP1	*yes*	HORVU.MOREX.r3.3HG0308890	Zm00001eb143690	Transcription factor viviparous 1	[135]
HvAHKs-1		HORVU.MOREX.r3.3HG0310920	At1g27320	Histidine kinase	[136]
HvRPT2A-2		HORVU.MOREX.r3.3HG0311570	At4g29040	26S protease regulatory subunit	[110]
HvARF4-1	*yes*	HORVU.MOREX.r3.3HG0312310	Os01g0927600	Auxin response factor	[83]
HvARF4-2		HORVU.MOREX.r3.3HG0313110	Os01g0927600	Auxin response factor	[83]
HvBDG1		HORVU.MOREX.r3.3HG0317920	Os11g0514400	Leucine-rich repeat receptor-like kinase	[137]
HvSNG1/HXK3		HORVU.MOREX.r3.3HG0318700	Os01g0940100	Phosphotransferase	[138]
Hvemp5		HORVU.MOREX.r3.3HG0323440	Zm00001eb140440	Pentatricopeptide repeat-containing protein	[139]
HvRGB1		HORVU.MOREX.r3.4HG0333750	Os03g0669100	Deoxyuridine 5'-triphosphate nucleotidohydrolase	[67]
Hvdek35	*yes*	HORVU.MOREX.r3.4HG0337450	Zm00001eb055010	Pentatricopeptide repeat-containing protein	[68]
HvAHKs-2	*yes*	HORVU.MOREX.r3.4HG0337770	At2g01830	Histidine kinase	[136]
Hvemp4	*yes*	HORVU.MOREX.r3.4HG0339220	Zm00001eb055980	Pentatricopeptide repeat-containing protein	[70]

HvDAR1		HORVU.MOREX.r3.4HG0341060	At4g36860	Protein DA1-related 1	[11]
HvTaSnRK2.10	*yes*	HORVU.MOREX.r3.4HG0342860	TraesCS4A02G235600	Kinase family protein	[140]
Hvsmk1		HORVU.MOREX.r3.4HG0345720	Zm00001eb110890	Pentatricopeptide repeat-containing protein	[141]
HvCyclin-T1;3-2		HORVU.MOREX.r3.4HG0350880	Os11g0157100	Cyclin family protein	[115]
HvSOD7/NGAL2-1		HORVU.MOREX.r3.4HG0351290	At3g11580	B3 domain-containing protein	[142]
HvEOD1/BB		HORVU.MOREX.r3.4HG0384230	At3g63530	RING/U-box superfamily protein	[143]
HvHDR3	*yes*	HORVU.MOREX.r3.4HG0389200	Os03g0267800	LIM domain-containing protein 1	[144]
HvANT-2		HORVU.MOREX.r3.4HG0394710	At4g37750	AP2-like ethylene-responsive transcription factor	[111]
HvTUD1	*yes*	HORVU.MOREX.r3.4HG0394810	Os03g0232600	U-box domain-containing protein	[145]
HvTaGS-4B	*yes*	HORVU.MOREX.r3.4HG0395540	TraesCS4B02G240900	Glutamine synthetase	[146]
HvD14-1		HORVU.MOREX.r3.4HG0398570	Os03g0203200	Sigma factor sigB regulation protein rsbQ	[147]
HvPGL1	*yes*	HORVU.MOREX.r3.4HG0403680	Os03g0171300	Transcription factor protein	[148]
HvFBK12-2		HORVU.MOREX.r3.4HG0403700	Os03g0171600	Kelch repeat-containing protein	[131]
Hvsmk2-1		HORVU.MOREX.r3.4HG0404270	Zm00001eb208970	Pyridoxal 5'-phosphate synthase subunit PdxT	[149]
HvBG1-2		HORVU.MOREX.r3.4HG0404460	Os03g0175800	Protein BIG GRAIN 1	[134]
HvANT-3		HORVU.MOREX.r3.4HG0404560	At4g37750	AP2-like ethylene-responsive transcription factor	[111]
HvLG3		HORVU.MOREX.r3.4HG0405650	Os03g0183000	Ethylene-responsive transcription factor	[150]
HvEXPLA1-1	*yes*	HORVU.MOREX.r3.4HG0409710	Os03g0132200	Expansin-like protein	[151]
HvEXPLA1-2		HORVU.MOREX.r3.4HG0409760	Os03g0132200	Expansin-like protein	[151]
HvEXPLA1-3		HORVU.MOREX.r3.4HG0409870	Os03g0132200	Expansin-like protein	[151]
HvCDPK1	*yes*	HORVU.MOREX.r3.4HG0410700	Os03g0128700	Calcium dependent protein kinase	[152]
Hvprog1-1		HORVU.MOREX.r3.4HG0411810	Os07g0153600	Zinc finger protein, putative	[153]
Hvprog1-2		HORVU.MOREX.r3.4HG0411820	Os07g0153600	Zinc finger protein, putative	[153]
Hvprog1-3		HORVU.MOREX.r3.4HG0411840	Os07g0153600	Zinc finger protein, putative	[153]
HvMRP5		HORVU.MOREX.r3.4HG0412040	Os03g0142800	Multidrug resistance protein ABC transporter family protein	[154]
HvCCS52A	*yes*	HORVU.MOREX.r3.4HG0412380	Os03g0123300	WD-repeat protein, putative	[155]
Hvprog1-4		HORVU.MOREX.r3.4HG0413540	Os07g0153600	Zinc finger protein, putative	[153]
Hvincw1-2		HORVU.MOREX.r3.4HG0413910	Zm00001eb242820	Cell wall invertase	[57]
HvIKU2-2		HORVU.MOREX.r3.5HG0421310	At3g19700	Receptor protein kinase, putative	[72]
HvPPKL3	*yes*	HORVU.MOREX.r3.5HG0426290	Os12g0617900	Serine/threonine-protein phosphatase	[73]
Hvcr4-1		HORVU.MOREX.r3.5HG0429770	Zm00001eb406780	Kinase family protein	[156]
HvDA1		HORVU.MOREX.r3.5HG0436650	At1g19270	Protein DA1-related 1	[157]
HvTaSnRK2.9-5A	*yes*	HORVU.MOREX.r3.5HG0438740	TraesCS5A02G069500	Kinase family protein	[158]
HvSOD7/NGAL2-2		HORVU.MOREX.r3.5HG0458770	At3g11580	B3 domain-containing protein	[142]
HvCEN2/RCN1	*yes*	HORVU.MOREX.r3.5HG0460080	Os11g0152500	Putative phosphatidylethanolamine-binding protein	[159]
Hvwri1	*yes*	HORVU.MOREX.r3.5HG0463120	Zm00001eb093560	AP2-like ethylene-responsive transcription factor	[160]
HvTTG2		HORVU.MOREX.r3.5HG0466860	At2g37260	WRKY transcription factor	[161]
HvBC12		HORVU.MOREX.r3.5HG0468240	Os09g0114500	Kinesin-like protein	[162]

HvDEP1	*yes*	HORVU.MOREX.r3.5HG0480200	Os09g0441900	Guanine nucleotide-binding protein subunit gamma 3	[163]
HvFBK12-3		HORVU.MOREX.r3.5HG0481140	Os03g0171600	Kelch repeat-containing protein	[131]
HvGS9	*yes*	HORVU.MOREX.r3.5HG0485690	Os09g0448500	Protein XRI1	[164]
HvSG1	*yes*	HORVU.MOREX.r3.5HG0486680	Os09g0459200	BEST plant protein match is: (TAIR:plant.1) protein, putative	[165]
Hvemp6	*yes*	HORVU.MOREX.r3.5HG0488360	Zm00001eb101330	Ubiquitin carboxyl-terminal hydrolase family protein	[166]
HvBC1	*yes*	HORVU.MOREX.r3.5HG0495090	Os09g0510500	basic helix-loop-helix (bHLH) DNA-binding superfamily protein	[167]
HvD14-2		HORVU.MOREX.r3.5HG0505590	Os03g0203200	Sigma factor sigB regulation protein rsbQ	[147]
HvGL3.1	*yes*	HORVU.MOREX.r3.5HG0508080	Os03g0646900	Serine/threonine-protein phosphatase	[115,168]
Hvemp10	*yes*	HORVU.MOREX.r3.5HG0510080	Zm00001eb057030	Pentatricopeptide repeat-containing protein, putative	[169]
HvMPPR6	*yes*	HORVU.MOREX.r3.5HG0512500	Zm00001eb058140	Pentatricopeptide repeat protein	[170]
HvANT-4		HORVU.MOREX.r3.5HG0513410	At4g37750	AP2-like ethylene-responsive transcription factor	[111]
Hvdek36		HORVU.MOREX.r3.5HG0515060	Zm00001eb213580	Ribose-5-phosphate isomerase A	[171]
HvDST		HORVU.MOREX.r3.5HG0516880	Os03g0786400	Zinc finger protein, putative	[76]
HvABA2		HORVU.MOREX.r3.5HG0524120	At1g52340	Short-chain dehydrogenase/reductase	[77]
HvNaPRT1	*yes*	HORVU.MOREX.r3.5HG0530510	Os03g0837300	Nicotinate phosphoribosyltransferase, putative, expressed	[172]
HvGL3.3	*yes*	HORVU.MOREX.r3.5HG0532220	Os03g0841800	Protein kinase	[83]
Hvemb14		HORVU.MOREX.r3.6HG0541050	Zm00001eb209870	Nitric oxide synthase 1	[173,174]
HvMADS87-4		HORVU.MOREX.r3.6HG0541730	Os03g0582400	AGAMOUS-like MADS-box protein	[103]
Hvsmk2-2		HORVU.MOREX.r3.6HG0552540	Zm00001eb208970	Pyridoxal 5'-phosphate synthase subunit PdxT	[149]
Hvdek10		HORVU.MOREX.r3.6HG0566240	Zm00001eb207310	Pentatricopeptide repeat-containing protein	[175]
HvSPL4	*yes*	HORVU.MOREX.r3.6HG0570860	Os02g0174100	Squamosa promoter-binding-like protein	[176]
HvMADS29	*yes*	HORVU.MOREX.r3.6HG0571720	Os02g0170300	MADS-box transcription factor	[177]
HvBT1	*yes*	HORVU.MOREX.r3.6HG0578050	Os02g0202400	Mitochondrial carrier family	[178]
HvCKX2-2		HORVU.MOREX.r3.6HG0579880	At2g19500	Cytokinin oxidase/dehydrogenase	[76,128]
HvCKX2-3		HORVU.MOREX.r3.6HG0579910	At2g19500	Cytokinin oxidase/dehydrogenase	[76,128]
HvGW2		HORVU.MOREX.r3.6HG0583520	Os02g0244100	Protein SIP5	[179]
HvFUWA		HORVU.MOREX.r3.6HG0585150	Os02g0234200	NHL repeat-containing protein	[180]
Hvubl1	*yes*	HORVU.MOREX.r3.6HG0599760	Zm00001eb249040	U6 snRNA phosphodiesterase	[181]
Hvnkd1-2	*yes*	HORVU.MOREX.r3.6HG0603120	Zm00001eb073830	Zinc finger protein, putative	[119]
HvOcl1		HORVU.MOREX.r3.6HG0603410	Zm00001eb126140	Homeobox-leucine zipper protein	[182]
Hvcr4-2		HORVU.MOREX.r3.6HG0604180	Zm00001eb406780	Receptor protein kinase, putative	[156]
HvRAV6	*yes*	HORVU.MOREX.r3.6HG0604470	Os02g0683500	B3 domain-containing protein	[183]
HvGS2	*yes*	HORVU.MOREX.r3.6HG0606810	Os02g0701300	Growth-regulating factor	[184]
Hvdek1		HORVU.MOREX.r3.6HG0608170	Zm00001eb014030	Calpain-like protein	[78]
HvLec1	*yes*	HORVU.MOREX.r3.6HG0611100	Zm00001eb253260	Nuclear transcription factor Y subunit B	[80]
HvTaGS1a	*yes*	HORVU.MOREX.r3.6HG0613270	TraesCS6A02G298100	Glutamine synthetase	[185]
HvPGL2	*yes*	HORVU.MOREX.r3.6HG0615060	Os02g0747900	Transcription factor protein	[148]
HvAHKs-3		HORVU.MOREX.r3.6HG0616300	At2g01830	Histidine kinase	[136]

HvSMG1		HORVU.MOREX.r3.6HG0622200	Os02g0787300	Protein kinase	[186]
Hvemb12	*yes*	HORVU.MOREX.r3.6HG0623680	Zm00001eb257720	Translation initiation factor IF-3	[187]
HvMADS87-5		HORVU.MOREX.r3.6HG0624300	Os03g0582400	AGAMOUS-like MADS-box protein	[103]
HvMADS87-6		HORVU.MOREX.r3.6HG0624320	Os03g0582400	AGAMOUS-like MADS-box protein	[103]
HvMADS87-7		HORVU.MOREX.r3.6HG0624330	Os03g0582400	AGAMOUS-like MADS-box protein	[103]
HvMADS87-8		HORVU.MOREX.r3.6HG0624340	Os03g0582400	AGAMOUS-like MADS-box protein	[103]
HvbHLH107	*yes*	HORVU.MOREX.r3.6HG0627040	Os02g0805250	basic helix-loop-helix (bHLH) DNA-binding superfamily protein	[188]
HvBLS1		HORVU.MOREX.r3.6HG0627710	Os02g0811000	LIGHT-DEPENDENT SHORT HYPOCOTYLS-like protein (DUF640)	[189]
HvFBK12-4		HORVU.MOREX.r3.7HG0635000	Os03g0171600	Kelch repeat-containing protein	[131]
HvFBK12-5		HORVU.MOREX.r3.7HG0635090	Os03g0171600	Kelch repeat-containing protein	[131]
HvD1		HORVU.MOREX.r3.7HG0641160	Os05g0333200	Guanine nucleotide-binding protein subunit alpha-like protein	[190]
HvDLT	*yes*	HORVU.MOREX.r3.7HG0642830	Os06g0127800	Scarecrow transcription factor family protein	[191]
Hvde18		HORVU.MOREX.r3.7HG0647150	Zm00001eb409250	Flavin-containing monooxygenase, putative	[192]
HvMAPK6	*yes*	HORVU.MOREX.r3.7HG0652970	Os06g0154500	Mitogen-activated protein kinase	[193]
Hvmrp1-2		HORVU.MOREX.r3.7HG0653280	Zm00001eb354390	MYB-related transcription factor	[100]
HvHGW	*yes*	HORVU.MOREX.r3.7HG0654600	Os06g0160400	Proline-rich cell wall protein-like protein	[194]
Hvmrp1-3		HORVU.MOREX.r3.7HG0654760	Zm00001eb354390	MYB-related transcription factor	[100]
Hvmrp1-4		HORVU.MOREX.r3.7HG0656920	Zm00001eb354390	MYB-related transcription factor	[100]
Hvmrp1-5		HORVU.MOREX.r3.7HG0656940	Zm00001eb354390	MYB-related transcription factor	[100]
Hvppr2263	*yes*	HORVU.MOREX.r3.7HG0659520	Zm00001eb374510	Pentatricopeptide repeat-containing protein	[195]
Hvsh1		HORVU.MOREX.r3.7HG0661420	Zm00001eb374090	Sucrose synthase	[196]
HvVRT-A2	*yes*	HORVU.MOREX.r3.7HG0664320	TraesCS7A02G175200	MADS box transcription factor	[30,197]
HvBU1	*yes*	HORVU.MOREX.r3.7HG0666110	Os06g0226500	Transcription factor protein	[198]
Hvmrp1-6		HORVU.MOREX.r3.7HG0666620	Zm00001eb354390	MYB-related transcription factor	[100]
HvWFP	*yes*	HORVU.MOREX.r3.7HG0679980	Os08g0509600	Squamosa promoter binding protein	[199,200]
HvERF115	*yes*	HORVU.MOREX.r3.7HG0681130	Os08g0521600	Ethylene-responsive transcription factor	[201]
HvGW8	*yes*	HORVU.MOREX.r3.7HG0684000	Os08g0531600	Squamosa promoter binding-like protein	[202]
HvWTG1/HvOTUB1	*yes*	HORVU.MOREX.r3.7HG0685210	Os08g0537800	Ubiquitin thioesterase	[203]
HvASP- LSL		HORVU.MOREX.r3.7HG0695720	Os08g0162100	Topless-related protein 1	[204]
HvTBP1		HORVU.MOREX.r3.7HG0697320	Os08g0174700	Receptor-like kinase	[205]
Hvcr4-3		HORVU.MOREX.r3.7HG0699810	Zm00001eb406780	Kinase family protein	[156]
HvFIE1		HORVU.MOREX.r3.7HG0702190	Os08g0137250	Fertilization independent endosperm 1 protein	[206]
HvCKX2-4		HORVU.MOREX.r3.7HG0716480	At2g19500	Cytokinin oxidase/dehydrogenase	[76,128]
HvTaMOC1	*yes*	HORVU.MOREX.r3.7HG0720900	TraesCS7B02G285500	GRAS transcription factor	[207]
HvMADS87-9		HORVU.MOREX.r3.7HG0723860	Os03g0582400	MADS-box transcription factor family protein	[103]
Hvemp7		HORVU.MOREX.r3.7HG0727380	Zm00001eb333280	Pentatricopeptide repeat-containing protein	[208]
HvGW6a	*yes*	HORVU.MOREX.r3.7HG0742750	Os06g0650300	N-acetyltransferase, putative	[209]
HvHOS59		HORVU.MOREX.r3.7HG0744200	Os06g0646600	Homeobox protein knotted-1, putative	[210]

HvBRD2	HORVU.MOREX.r3.7HG0749940	Os10g0397400	Delta(24)-sterol reductase	[211]
HvMADS87-10	HORVU.MOREX.r3.7HG0751440	Os03g0582400	MADS-box transcription factor family protein	[103]
